# Health-related quality of life among patients with rheumatoid arthritis in Zanzibar: a prospective cohort study

**DOI:** 10.1007/s11136-025-03974-3

**Published:** 2025-05-07

**Authors:** Sanaa S. Said, Kjell Arne Johansson, Tone Wikene Nystad, Johnson Jeremia Mshiu, Bjorg-Tilde Svanes Fevang

**Affiliations:** 1 School of Health and Medical Sciences, The State University of Zanzibar, Zanzibar, Tanzania; 2 Department of Global Public Health and Primary Care, Bergen Center for Ethics and Priority Settings (BCEPS), Bergen, Norway; 3 Department of Clinical Science, University of Bergen, Bergen, Norway; 4 Department of International Collaboration (DIC), Haukeland University Hospital, Bergen, Norway; 5 Department of Internal Medicine, Mnazi Mmoja Hospital, Zanzibar, Tanzania; 6 Department of Addictive Medicine, Haukeland University Hospital, Bergen, Norway; 7 Department of Rheumatology, Haukeland University Hospital, Bergen, Norway; 8 National Institute for Medical Research, Muhimbili Research Center, Dar Es Salaam, Tanzania

**Keywords:** Rheumatoid arthritis, Quality of life, DMARD, Zanzibar, Visual analog scale

## Abstract

**Purpose:**

Rheumatoid arthritis (RA) is a chronic inflammatory illness that mainly affects the joints. Untreated, it causes deformity, poor health-related quality of life (HRQoL) and increased morbidity and mortality. There have been tremendous strides in RA therapies globally but with sparse availability in resource-limited settings. We aimed to evaluate HRQoL among patients with RA and its related factors.

**Methods:**

132 patients with RA were enrolled and followed up for one year. The same number of healthy controls were recruited. HRQoL was assessed using the EuroQoL five-dimension five-level tool (EQ-5D). From the responses, utility and visual analog scale (VAS) scores were obtained. Analysis of variance and independent t tests were used to compare the utility and VAS scores for patient subgroups. Factors influencing HRQoL were investigated through multiple linear regression analysis. A p value of < 0.05 was considered significant.

**Results:**

At baseline, mean patient utility score was 0.50 ± 0.06 and increased to 0.66 ± 0.10 at one year (p < 0.05). Compared to controls, whose mean utility score was 0.93 ± 0.02, patients with RA at one year had lower HRQoL (p < 0.001). Time to diagnosis of ≥ 1 year and higher disease activity were associated with poorer HRQoL. Patient VAS scores also improved from baseline to one year but were significantly lower than controls.

**Conclusion:**

HRQoL of patients with RA was lower than in controls but improved at one year indicating the impact of treatment. Reducing delays in diagnosis and initiating early and aggressive treatment may help to improve the HRQoL.

**Supplementary Information:**

The online version contains supplementary material available at 10.1007/s11136-025-03974-3.

## Plain English summary

Patients with rheumatoid arthritis have poorer quality of life compared to healthy controls. Data on this in sub-Saharan Africa is sparse and few studies have used controls for comparison. In this study we aimed to assess the quality of life of patients with RA, factors that affect it and compare them to controls. Patients were followed up for one year and we also assessed the quality of life before and after treatment. We found the dimension most affected among patients at baseline was pain or discomfort and the least affected was anxiety or depression. Patients who had a delay in diagnosis and active disease had poorer quality of life. Overall, patients with rheumatoid arthritis had lower quality of life compared to controls. The quality of life improved after one year of treatment but did not match that of the controls. Treating patients with rheumatoid arthritis can improve their quality of life particularly if treatment is initiated early and is focused on reducing disease activity.

## Introduction

Rheumatoid arthritis (RA) is a chronic inflammatory disorder that mainly affects the joints. Global prevalence of RA is estimated to be 0.21% [1]. Data for RA in sub-Saharan Africa is sparse but estimates give a prevalence of 0.096 [1]. There are no national surveys for Tanzania, but the global burden of disease estimates a prevalence of RA of 0.094% [1]. Specific data for Zanzibar are lacking.

When left untreated, RA can lead to joint deformities and increased morbidity and mortality. A vast improvement in treatment, including the implementation of new treatment strategies as well as several disease- modifying anti-rheumatic drugs (DMARDs), has improved disease specific outcomes and health-related quality of life (HRQoL) for patients with RA [1–3]. Despite advances in RA treatment, there are large global inequalities in the availability, access, and affordability of newer treatments, and patients in low-income countries have limited access to and availability of essential RA care [4]. Despite advances in care, RA has a negative impact on almost all aspects of patients’ lives [5–7], and these patients are reported to have lower HRQoL than their age- and sex-matched controls [8] as well as patients with other chronic illnesses [8, 9].

The World Health Organization (WHO) defines quality of life as “an individual’s perception of their position in life in the context of the culture and value systems in which they live and in relation to their goals, expectations, standards and concerns” [10]. There are several generic tools used to measure HRQoL, such as the EuroQoL 5-dimension 5-level (EQ-5D), short-form 36 (SF-36), and World Health Organization quality of life (WHOQOL) questionnaires [11].

In an RA cohort from India, findings revealed lower levels of social support and higher levels of stress, anxiety and depression than in healthy individuals [12]. In Egypt, all aspects of HRQoL were affected in those with RA compared with healthy age- and sex-matched controls [13]. Compared with patients with hand osteoarthritis, patients with RA had worse general health, physical function and mental health [14]. Factors that have been associated with decreased HRQoL include severe disease activity, increasing age, female sex, functional disability, comorbidity and disease duration [15, 16]. HRQoL is also affected by socioeconomic factors such as employment, economic status and lifestyle habits such as physical activity, alcohol, smoking and diet [16, 17]. Studies have suggested that there may be geographical differences in HRQoL among patients with the same disorders [18]. Standard disease-modifying antirheumatic drugs can significantly improve all domains of HRQoL in patients with RA [19].

There is sparse data addressing HRQoL in patients with RA from sub-Saharan Africa. This may be due to the majority of HRQoL questionnaires being developed in high-income countries. To date, no studies have assessed HRQoL in patients with RA in Tanzania mainland or Zanzibar where access to rheumatology care may be limited. Our study aimed to assess HRQoL among patients with RA in Zanzibar compared with controls and analyze the factors that influence it.

## Methods

### Study design, setting and population

We conducted an observational prospective cohort survey. All patients ≥ 18 years with a diagnosis of RA attending the rheumatology outpatient clinic at the main referral hospital in Zanzibar (Mnazi Mmoja Hospital) were invited to participate. This is the only rheumatology clinic in Zanzibar providing DMARD therapy. Data from 132 patients was collected from September 2019 to August 2023.

An interview and physical examination were carried out on all patients by the principal investigator (PI). For the HRQoL, the EQ-5D Swahili version was provided; it was self-administered for the literate and able participants, and interview-guided questionnaires were provided for those who were not. Patient records were also assessed. Data was collected on paper forms and later entered in the Vervig® database.

We also collected similar HRQoL data from 132 healthy controls. They consisted of relatives accompanying patients to the hospital obtained from various outpatient clinics other than the rheumatology clinic. We used convenient sampling and those who were ≥ 18 years of age and reported no history of preexisting medical conditions were included. Due to limitations of resources, we did not match age, gender or socio-economic status. For this reason, some differences in age and gender distribution were present, and this is described in Table 1. To reduce potential confounding we did not enlist relatives of patients with RA. Data from controls was collected only once. All controls provided informed consent.

### Study variables

From the patient interviews, we collected sociodemographic data (age, sex, disease duration at enrollment, marital status, educational level and smoking history) and data regarding HRQoL, total household and health care expenditure, and medication adherence via the 19-item compliance questionnaire rheumatology (CQR19). We also added a set of locally relevant questions to assess medication adherence: when was the last time you took your medication? Was it on time? Was the dose correct? Did you ever run out of medication? Did you ever forget to take your medication?

Information on monthly total household expenditure was collected at baseline and included expenses for food, clothing, rent, utilities, education, health insurance, household utensils and repairs, travel costs, loan repayment and other expenses. These were collected in Tanzanian shillings and converted into United States dollars (USD) via the 2022 conversion rate.

Patient records were assessed to determine preexisting medical conditions. These include cardiovascular disease, endocrine disorders, neurological disorders, renal disorders, chronic infections, chronic respiratory disease, inflammatory back pain, malignancy, liver disease, skin disease, uveitis and inflammatory bowel disease. We also recorded erythrocyte sedimentation rates (ESR) and previous rheumatic drug use. Data on radiological damage was obtained from radiologist reports and was classified as positive if it contained any features of RA-related changes (juxta-articular osteopenia, joint space narrowing, uniform cartilage destruction and erosive changes).

The physical examination included a general assessment and a 28-joint assessment to determine disease activity, which was calculated using the clinical disease activity index (CDAI). A score of > 22 is defined as high disease activity, ≤ 22 to > 10 moderate disease activity, ≤ 10 to > 2.8 low disease activity, and ≤ 2.8 as remission.

Data was collected at all visits, but only data at baseline and one year (9 to 15 months) after inclusion, was used for the present study. At one year, data was available for 78 patients.

### Health-related quality of life instrument

The EuroQol 5D-5L is an internationally used measure of HRQoL validated for use in RA [20]. It has been validated for use in resource limited settings (ref) and a Swahili version of the questionnaire is provided by EuroQol. It contains self-reported questions on five dimensions of health (mobility, self-care, usual activities, pain/discomfort and anxiety/depression) graded into five levels ranging from no problems, slight problems, moderate problems, severe problems to extreme problems. Patients indicate the level that corresponds to the day of completion of the questionnaire. A patient in the best and worst health in all the domains would score 11,111 and 55,555 respectively. This figure is converted into a single index utility score (0 to 1, where 0 is worst and 1 is best health, values < 1 represent states considered worse than death) based on weights per dimension from population-based preference surveys. In this analysis, we used weights of value sets from Uganda [21] as there is currently no value set developed for Tanzania. The tool also contains a visual analog scale (VAS), which evaluates self-perceived patient overall health from 0 (worst imaginable health) to 100 (best imaginable health).

### Data management and statistical analysis

Data management and analysis were conducted via Stata version 17 (Stata Corp, Texas, USA). Descriptive analyses were employed to generate summary statistics. Continuous variables were presented as means and standard deviations, whereas categorical variables were presented as frequencies and percentages. Pearson’s chi-square test was used to determine the associations between the characteristics of the patient versus control group.

Responses to the EQ-5D questionnaire for both patients and controls were summarized via bar graphs. Paired and independent t-tests were utilized to compare the mean scores for both utility and the VAS at patient baseline and year one versus controls. Boxplots were used to compare median HRQoL utility and VAS scores for patients at baseline, year one and for controls. We also compared the mean utility scores for each of the dimensions.

Patients were divided into subgroups according to their sociodemographic and clinical characteristics. The normality of continuous variables was assessed using Q-Q plots of residuals following an ANOVA test as well as by directly testing the specific variable before conducting a two-sample independent t-test or paired t-test. We used ANOVA and independent t tests to compare the mean utility and VAS scores across the groups using combined baseline and year one scores. We assessed for potential confounders using a directed acyclic diagram (DAG) (Supplement 1). Factors influencing HRQoL were investigated through multiple linear regression analysis and all variables were included in the multivariable analysis due to their clinical importance to the study. The 95% confidence interval (CI) was calculated for each coefficient, and results were deemed significant at p < 0.05.

## Results

The majority of patients were female (86%). The mean age was 45 ± 13 years. On average, patients had a long disease duration and presented with moderate disease activity at baseline. For the controls, majority were also female (71%) with a mean age of 43.7 ± 11 years and overall higher level of education than the patients (Table 1).

**Fig. 1:**
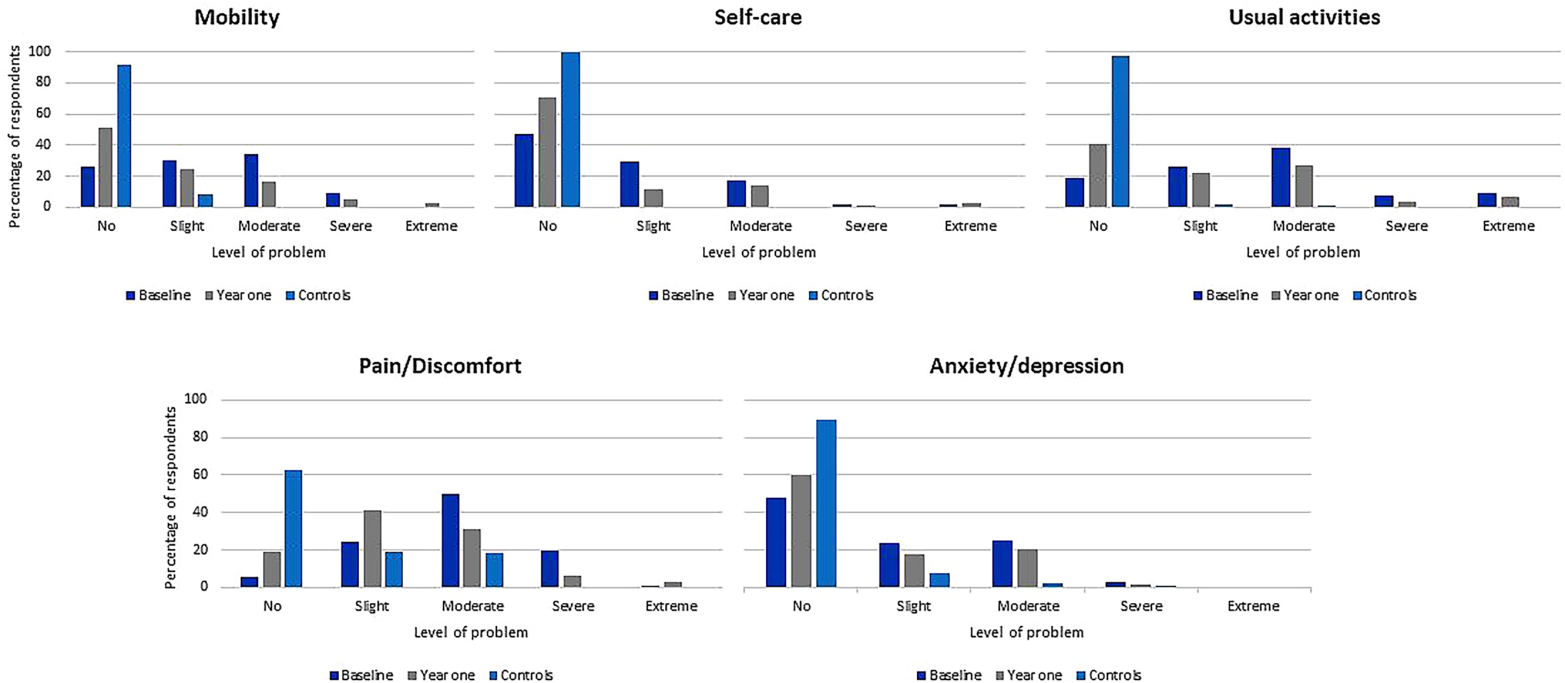
EQ-5D responses for patients at baseline, patients at 1-year follow-up, and controls categorized according to dimension and level. A total of 132, 78 and 132 participants were available at baseline, one-year and for the control group respectively. The responses were categorized into levels of severity of problems for each of the dimensions from no problem to extreme problems. These were then plotted according to level of severity for comparison between the groups

**Fig. 2:**
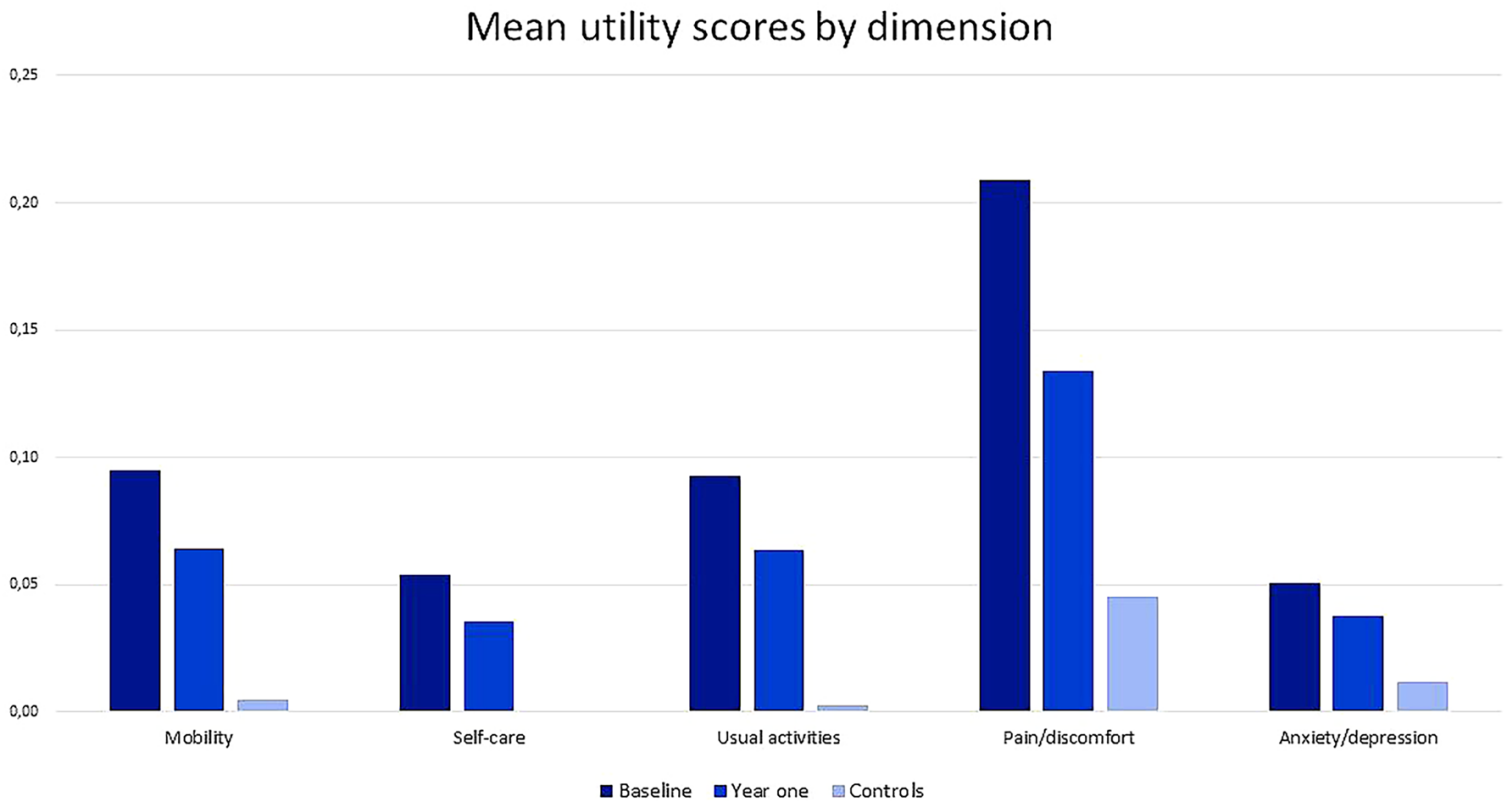
EQ-5D mean utility scores for each dimension for the different groups after conversion using the Uganda normal population value set. A total of 132, 78 and 132 participants were available at baseline, one-year and for the control group respectively. We converted the EQ-5D responses for the different dimensions into a mean single digit with 0 indicating best health

Figure 1 shows the EQ-5D scores for each dimension in the patients with RA at baseline and at one year as well as in the controls. The best health score (11,111) was found in only 5 patients (3.8%) at baseline, 12 patients at 1 year (15%) and 54 controls (51%). None of the respondents reported the worst health (55,555). We found that self-care and usual activities had the strongest positive correlation of 0.73 while anxiety or depression and pain or discomfort had the weakest positive correlation. There was no negative correlation between the dimensions (Supplement 2). The mean utility scores revealed that pain/discomfort was the dimension most affected in all the groups. The least affected dimension for patients at baseline was anxiety and depression. For patients at one year and controls, the least affected dimension was self-care (Fig. 2).

**Table 1:** Characteristics of patients and controls

Variable	Cohort n = 132	Controls n = 132	p value
Sex (female, %)	114 (86)	94 (71)	0.003
Age, years (mean, SD)	45.2 ± 13	43.7 ± 11	0.308
Disease duration^1^, years (median IQR)	4 (2–6)	NA	
Time to diagnosis^2^, years (median IQR)	1.5 (1–4)		
Erythrocyte sedimentation rate at baseline (mean, SD)	46 ± 34	NA	
Erythrocyte sedimentation rate at 1 year (mean, SD)	38 ± 30	NA	
Clinical disease activity score at baseline (mean, SD)	18.3^3^ ± 12.6	NA	
Clinical disease activity score at 1 year (mean, SD)	8.6^4^ ± 8.4	NA	
Preexisting medical conditions^3^ (yes, %)	51 (39)	NA	
Marital status (number, %):			
Single	16 (12)	NA	
Married	84 (64)		
Widowed	8 (6)		
Divorced	24 (18)		
Education level: (number, %)			0.037
None	16 (12)	5 (4)	
Primary	33 (25)	29 (22)	
Secondary	65 (49)	83 (63)	
Tertiary	18 (14)	15 (11)	
Smoking history: (number, %)			
Never	120 (91)	NA	
Ever smoker^4^	12 (9)		
Radiological damage^5^ (yes, %) *	65 (64)	NA	
Total monthly household expenditure in USD (mean, ± SD)	250 ± 135	NA	
Medication adherence at 1 year (yes, %)	49 (64)	NA	
Medication type at 1 year			
csDMARDs	73 (94)	NA	
bDMARD	5 (6)		

p values were calculated using chi-square test

*SD* standard deviation, *IQR* interquartile range, *NA* not applicable, *csDMARDs* conventional synthetic disease-modifying antirheumatic drugs, including methotrexate, sulfasalazine, hydroxychloroquine and leflunomide, *bDMARDs* biological disease-modifying antirheumatic drugs, specifically rituximab

^1^Disease duration indicates the time from first symptoms to enrollment

^2^Time to diagnosis indicates the time from first symptoms to when RA diagnosis was made

^3^Preexisting medical conditions, including cardiovascular disease (hypertension, congestive heart failure, myocardial infarction, and peripheral vascular disease), endocrine disorders (diabetes and thyroid disorders), neurological disorders (stroke and dementia), renal disorders, chronic infections, chronic respiratory disease, inflammatory back pain, malignancy, liver disease, skin disease, uveitis and inflammatory bowel disease

^4^ Ever smoker, includes both past and current smokers

^5^Radiological damage—features of RA-related changes (juxta-articular osteopenia, joint space narrowing, uniform cartilage destruction and erosive changes) visualized via radiological imaging

^*^30 missing

### Comparison of health-related quality of life between patients with RA and controls

For the mean utility scores, there was a notable improvement from baseline to year one from 0.47 ± 0.08 to 0.66 ± 0.10 (*p* = 0.003). Similarly, the mean VAS score also significantly increased from a mean of 52.7 ± 3.7 to 74.3 ± 4.0 from baseline to year one (p < 0.001). The utility and VAS scores for patients at baseline and year one were significantly lower than those of the control group, whose mean utility score was 0.93 ± 0.02 (p < 0.001 for patients at one year vs controls) and VAS score was 86.7 ± 2.7 (*p* < 0.001 for patients at one year vs controls).

**Fig. 3:**
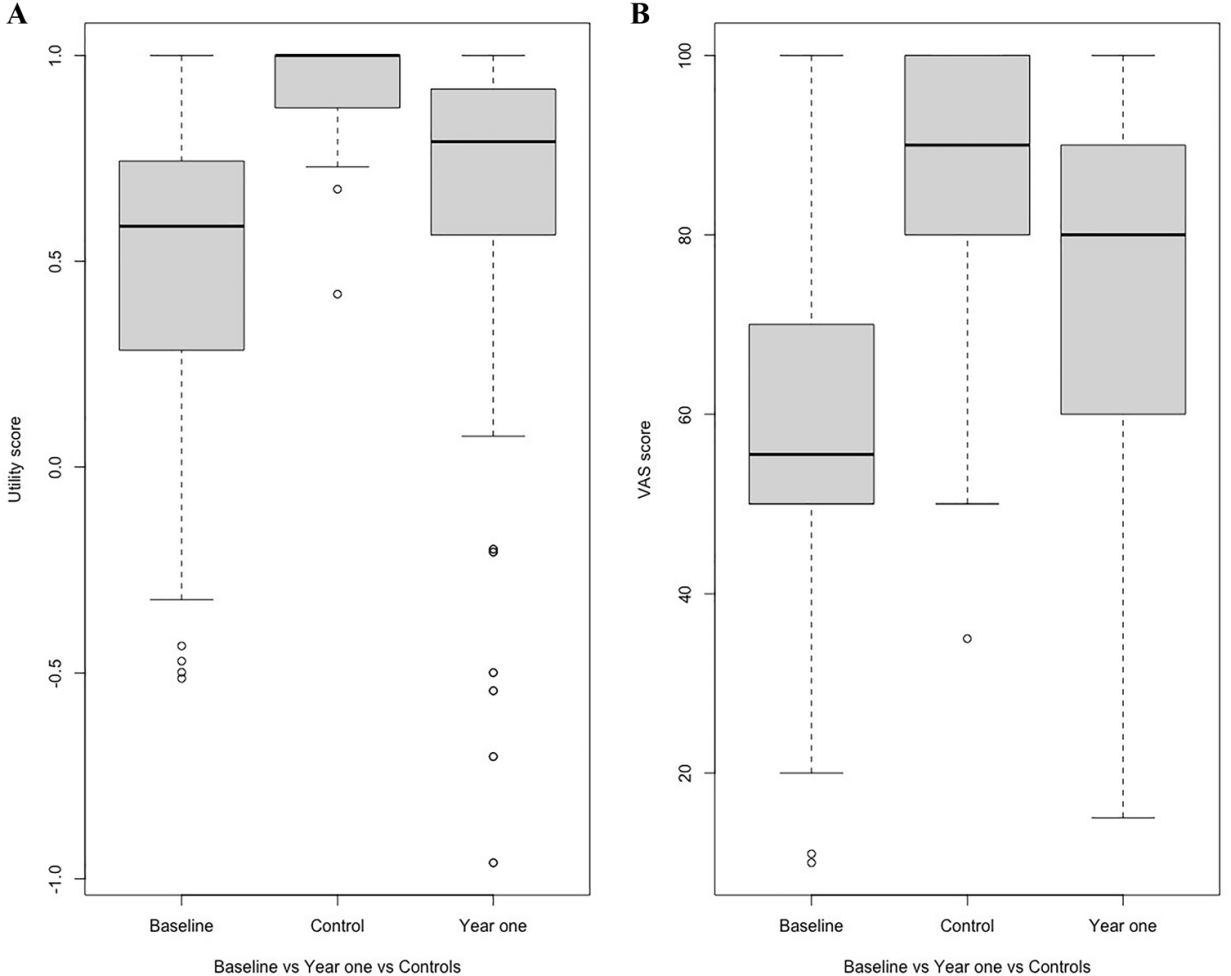
Comparison of health-related quality of life between patients with RA (baseline and at one year) and controls via the EQ-5D utility score and visual analog scale (VAS). The box plot consists of the horizontal line within each box denoting the median value, whereas the ends of the box denote the 25th and 75th distributions of the values for each group. The vertical dotted lines represent data from the minimum value to the 1st quartile and the 3rd quartile to the maximum value. The dots represent data outside the range of the adjacent values (outliers). For the utility scores (**A**) patients at baseline had the lowest scores and showed significant improvement at year one. The controls exhibited high utility scores. For the VAS (**B**), the median VAS also improved significantly from baseline to year one while the controls had a high median VAS score

At baseline, the median utility score was approximately 0.58 (Fig. 3). By year one, there was significant improvement, to approximately 0.79, whereas the control group consistently exhibited high utility scores, with a median of 1. The median VAS score significantly improved from 55.5 to 80.0 from baseline to year one. The control group maintained a consistently high median VAS score of 90 (Fig. 3).

### Factors influencing HRQoL scores in patients with RA

The assessment of HRQoL among patients with RA using combined baseline and year one utility and VAS scores revealed several notable findings (Table 2). Patients on conventional synthetic disease-modifying antirheumatic drugs (csDMARDs) presented numerically higher mean utility scores (0.58 ± 0.3) than did those on biologic DMARDs (bDMARDs) (0.35 ± 0.41, p = 0.072), although the mean VAS scores did not differ between the two groups. Patients with radiological damage had both lower utility (0.51 ± 0.41, p = 0.035) and VAS scores (61.5 ± 21.9, p = 0.013) than did those without radiological damage. There was a statistically significant difference in HRQoL for the different levels of disease activity as measured by the CDAI using both utility and VAS scores (Table 2).

**Table 2:** Comparison of health-related quality of life among rheumatoid arthritis patient groups using utility and visual analog scale scores

Variable	Total	Utility score	*p* value	VAS score	*p* value
		mean ± SD		mean ± SD	
Sex					
Male	24	0.58 ± 0.44	0.825	67.74 ± 13.12	0.433
Female	186	0.56 ± 0.38		64.29 ± 20.52	
Medication					
csDMARDs	140	0.58 ± 0.39		63.81 ± 20.40	
bDMARDs	10	0.35 ± 0.41	0.079	61.5 ± 12.70	0.725
Comorbid					
No	130	0.58 ± 0.36		63.86 ± 20.02	
Single	53	0.55 ± 0.40	0.315	67.55 ± 19.15	0.462
More than one	25	0.46 ± 0.46		62.72 ± 21.24	
Smoking history					
Nonsmokers	193	0.57 ± 0.38	0.343	64.83 ± 20.17	0.708
History of smoking	17	0.47 ± 0.42		62.9 ± 16.01	
Adherence					
No	133	0.59 ± 0.35	0.118	65.28 ± 20.44	0.562
Yes	77	0.50 ± 0.45		63.62 ± 18.83	
Age group					
18–39 years	71	0.51 ± 0.40		61.77 ± 20.41	
40–59 years	111	0.60 ± 0.36	0.307	66.00 ± 19.79	0.320
≥ 60 years	28	0.54 ± 0.42		66.71 ± 18.41	
Time to diagnosis					
< 1 year	102	0.60 ± 0.36		65.87 ± 20.95	
≥ 1 year	108	0.53 ± 0.40	0.168	63.54 ± 18.76	0.399
Disease duration					
< 1 year	24	0.59 ± 0.39	0.613	80.96 ± 11.70	< 0.001
≥ 1 year	186	0.56 ± 0.38		62.55 ± 19.72	
Clinical damage					
No	67	0.49 ± 0.41	0.069	65.72 ± 21.52	0.602
Yes	143	0.59 ± 0.37		64.18 ± 19.05	
Radiological damage					
No	60	0.65 ± 0.34	0.035	69.88 ± 17.43	0.013
Yes	105	0.51 ± 0.41		61.52 ± 21.85	
Level of education					
Primary	51	0.49 ± 0.39	0.551	64.04 ± 18.55	0.721
Secondary	102	0.58 ± 0.40		66.15 ± 19.97	
Tertiary	29	0.58 ± 0.35		61.65 ± 22.07	
No formal education	28	0.60 ± 0.32		63.68 ± 19.85	
CDAI					
Remission	31	0.81 ± 0.29	< 0.001	80.58 ± 20.25	< 0.001
Low	66	0.72 ± 0.24		68.69 ± 19.84	
Moderate	65	0.50 ± 0.31		58.58 ± 17.45	
High	48	0.25 ± 0.46		57.29 ± 15.29	

For analysis of comorbid conditions, age groups, level of education and CDAI analysis of variance (ANOVA) were used. The t-test was used for analysis of medication, smoking history, adherence, time to diagnosis, disease duration and radiological damage

The analyses used a combination of both baseline and year one scores

*VAS* visual analog scale, *csDMARDs* conventional synthetic disease-modifying antirheumatic drugs, *bDMARDs* biologic disease-modifying antirheumatic drugs, *CDAI* clinical disease activity index

### Determinants of HRQoL as measured by utility and VAS score among patients with RA

Using linear regression analysis, we found that none of the sociodemographic factors had a significant effect on HRQoL utility scores (Table 3). For the clinical factors, a time to diagnosis of one year or longer had a negative effect on the utility score (coefficient: −0.458, p = 0.013). The use of bDMARDs was also associated with lower utility scores (coefficient: −1.266, p = 0.012). Moderate and high CDAI values were associated with lower utility scores (coefficients: −0.313, −0.738; p = 0.025, 0.004, respectively) (Table 3). Only factors showing a p value of < 0.1 are displayed in Table 3. The complete analysis results are presented in Supplement 3. Among the sociodemographic characteristics, we found that only household expenditure was associated with the VAS score (coefficient: −0.394, p = 0.002) (Table 3). For every increase in household expenditure of one USD per month there was a decrease in VAS score of 0.394. Time to diagnosis of ≥ 1 year was associated with lower VAS scores (coefficients: −0.532, p = 0.038). Moreover, CDAIs of low, moderate, and high disease activity were associated with lower VAS scores (coefficients: −0.832, −1.583, and −1.686; p = 0.001, 0.000, and 0.000, respectively) compared to being in remission. The complete analysis results are presented in Supplement 4.

**Table 3:** Determinants of health-related quality of life using multiple linear regression analysis for utility and VAS scores

Variable	Coeff	Std error	95% CI	*p* value
Utility Score				
Sex				
Female	−1.047	0.589	−2.225; 0.131	0.080
Time to diagnosis				
≥ 1 year	−0.458	0.180	−0.819; −0.098	0.013
Medication				
bDMARDs	−1.266	0.488	−2.242; −0.29	0.012
Comorbid conditions				
1	0.301	0.157	−0.014; 0.615	0.061
> 1	0.135	0.202	−0.269; 0.538	0.507
CDAI				
Low	−0.215	0.173	−0.560; 0.131	0.219
Moderate	−0.709	0.191	−1.090; −0.328	< 0.001
High	−0.750	0.237	−1.224; −0.276	0.002
VAS score				
Age	0.018	0.010	−0.002; 0.039	0.077
Household expenses (USD)	−0.394	0.120	−0.634; −0.154	0.002
Time to diagnosis				
≥ 1 year	−0.532	0.251	−1.033; −0.03	0.038
CDAI				
Low	−0.832	0.248	−1.327; −0.336	0.001
Moderate	−1.583	0.273	−2.129; −1.037	< 0.001
High	−1.686	0.341	−2.367; −1.005	< 0.001

Comparison subgroups are as follows: sex – male, time to diagnosis −< 1 year, medication -conventional synthetic disease-modifying antirheumatic drugs, comorbid conditions – none, CDAI – remission

*Coeff* regression coefficient, *Std err* standard error, *CI* confidence interval, *bDMARDs* biologic disease-modifying antirheumatic drugs, *CDAI* clinical disease activity index, *VAS* visual analog scale, *USD* United States dollar

## Discussion

The major finding of this study was that the health-related quality of life was lower in patients with RA than in controls. Although it improved from baseline to one year, among RA-patients, the quality of life was still lower than in the controls. Additionally, those with severe disease, as demonstrated by the presence of radiological damage, had a lower HRQOL. On the positive side, the HRQoL improved at one year, indicating that treatment had an impact on life quality. Similarly, compared with patients in remission, those with low, moderate and high disease activity had poorer HRQoL, indicating that achieving remission is likely to improve HRQoL.

High disease activity using different measures was found to be associated with reduced HRQoL in several studies [7, 22–26]. The findings concerning the association between radiological damage and HRQoL have been conflicting, with some showing reduced HRQoL, whereas others have shown no correlation [25, 27–29]. We found it to be significant in the separate analyses, possibly indicating some importance but not strong enough to be detected in the multiple regression. Findings on the associations between bDMARDs and HRQoL are also variable [30–34]. We found that bDMARD use was associated with poorer HRQoL. This may be attributed to our treatment protocol recommending bDMARDs only after a lack of improvement on csDMARDs which indicates that they were only used in patients with severe disease. Data is however, conflicting. In studies where bDMARDs were used much earlier and before failure of csDMARDs HRQoL improved significantly [35]. In a German bDMARD registry study for patients with longstanding RA, Gerhold et al. found that HRQoL improved even among patients with sequential bDMARD therapy. Although patients’ HRQoL was much lower than the general population in this study, lasting improvements were obtained even with sequential bDMARD therapy [36]. Our results should therefore be interpreted with caution particularly because of the low number of patients who were on bDMARDs in our cohort.

In our cohort, we found that pain and discomfort was the dimension most affected, similar to several other studies [15, 22, 29, 35]. At baseline, anxiety or depression was reported to be the least affected, with no report of extreme problems and the majority reporting no problem. This is expected, as RA commonly presents with pain and swelling of the joints. This may subsequently affect mobility, self-care and daily activities, perhaps more than mental health.

Several studies have shown an association of age, comorbid diseases and female sex with HRQoL [6–8]. Our findings did not reflect this. Compared with other RA populations with a mean age of 58 to 60 years [28, 36], our patient group was relatively young, with a mean age of 45.2 years and a small age range possibly not sensitive enough to detect the change in HRQoL associated with aging. Similarly, comorbid conditions were present in only 39% of our patients. The most common condition was hypertension, which is considered symptomless and may have had little impact on patient HRQoL. We did not find a gender effect, perhaps due to the low number of male participants in the study.

A longer disease duration has been associated with poorer HRQoL in a number of studies [6, 13, 25]. In our study, we found an association between time to diagnosis and HRQoL via both the VAS score and the utility score. Patients with a delay in diagnosis subsequently have more difficult-to-treat RA [37], which may explain the association with poorer HRQoL. Interestingly, Alotaibi et al., while assessing HRQoL in Saudi Arabia, did not find any associations with age, sex, comorbidities, disease or treatment duration [38]. We found a lower HRQoL associated with increasing household expenditure. Perhaps this could be related to the fact that patients with worse disease were more likely to be unable to carry out activities such as farming and would have to purchase foods or require to employ help for their household chores.

Overall, patients with RA have been found to have a lower HRQoL than the general population [5, 6, 8, 12, 13]. Compared with other patient groups, patients with RA have lower HRQoL than those with PsA, AS, osteoarthritis, diabetes mellitus, hypertension, myocardial infarction or clinical depression [6, 8, 9, 14, 39]. In agreement with other similar studies across Africa, patients with RA are affected in all aspects of HRQoL [5, 13, 25, 40]. Our findings are also similar to those reported in meta-analysis from Asia, which reported a mean utility score of 0.60 ± 0.06 in cohorts of patients with RA. The same meta-analysis reported a pooled VAS score of 61.2 ± 10.48, which was lower than 74.0 ± 17.6 in our cohort [7].

Across Europe, the HRQoL of patients with RA is much lower, with utilities ranging from 0.43 to 0.62 [36, 41–43]. They also noted utility scores of less than 0, indicating conditions considered worse than death, both at baseline and follow-up [41, 44]. In the United Kingdom, Scott et al. reported that patients with RA did not achieve the same HRQoL as age- and sex-matched controls, despite the availability of newer therapies and intense treatment, and even after achieving remission. They compared patients with both early and established RA, and although remission optimized HRQoL, it did not normalize it [34]. Using global burden of disease (GBD) 2021 disability weight data, patients with mild, moderate and severe RA would have utility scores of 0.883, 0.683 and 0.419 [45], respectively, which are much higher than those found in our patient population.

Although the EQ-5D has been validated for use in RA, some cultural and societal aspects must be considered, and caution should be exercised during interpretation. Anxiety and depression were the least common symptoms among our patients, despite studies from other regions indicating that it is commonly affected in patients with chronic RA [22, 34]. Prevalence of depression and anxiety in patients with RA is reported to be around 17% [46, 47]. Patients with depression have also been reported to be at risk of developing RA [48]. Along the Swahili coast, Zanzibar included, mental health is commonly considered an ailment to be treated by traditional methods and not Western medicine [49]. In particular, the word ‘wasiwasi’ used in the Swahili language to mean anxiety is often associated with the Quranic interpretation of ‘whisperings of the devil’ and has a negative connotation [50]. In turn, it is not considered serious enough to be worth reporting to a medical doctor and patients opt to seek religious or spiritual healers to help them deal with these symptoms [49]. Lastly, it is considered taboo to complain of one’s illness, which is believed to be a test from God, and bearing it stoically as acceptance of divine will [51]. Analysing life quality within a particular cultural setting is meaningful but care must also be taken when comparing HRQoL findings across cultures as different age groups, cultures and settings consider different aspects important to HRQoL. To overcome the biases that may be created particularly by the under-reporting of anxiety and depression, future researchers may consider using additional locally validated tools specifically assessing depression and/or anxiety. This will allow detection of any reporting differences between depression specific tools and anxiety and depression within the EQ-5D questionnaire.

HRQoL is an important aspect of healthcare but is not part of routine assessment in Zanzibar. To clinicans and policymakers, it may reveal various factors that affect overall patient health. The incorporation of HRQoL measurements in routine care may lead to improved service quality and overall HRQoL for patients [12]. It can also potentially provide evidence on areas for health-spending to improve outcomes. Patients with early RA who receive intensive treatment are more likely to achieve and sustain remission [52]. Educational programs to increase RA awareness could mitigate diagnostic delays. Early diagnosis and aggressive treatment are measures that can be improved locally and subsequently improve HRQoL.

Our study used the Uganda EQ-5D value set for utility calculations as Tanzania-specific value-sets are not available. Other value sets for the EQ-5D-5L from EuroQoL for African populations are from Egypt and Ethiopia. We felt that the Uganda value set would be more representative of the Zanzibar population as they are both countries in East Africa with some similarities. These include similar background in terms of colonialism and healthcare structures, the importance of the family and community in the care for the sick as well as belief in spiritual and alternative healing [53]. The Egyptian value-set is considered of value for the Middle Eastern and North African region due to their cultural similarities. We do acknowledge however that some religious, cultural and socioeconomic differences still exist between Uganda and Tanzania and may have impacted our results. Of particular note is the robust mental health policy in Uganda with integration into primary health care [54] which in comparison is still under improvement in Zanzibar.

Our study is unique in this setting and presents new findings which are of importance as to the influence of RA on patients’ lives. There are few comparable studies in sub-Saharan Africa, and none of them use a control group for comparison. Our control group consisted of randomly selected relatives of patients attending outpatient clinics. Due to this, they were slightly younger than the patient group, as in our setting, it is more likely that a younger adult relative would accompany a patient to hospital than an elderly person. We also lacked some possibly relevant information for the controls, such as marital status and household expenditure, which may have affected the HRQoL. Despite these weaknesses, we believe comparing to a control group chosen from the same setting adds valuable information and our study consequently presents novel findings for the African context. Additionally, because of the nature of data collection, one cannot completely rule out self-reporting bias whether from social desirability or recall bias when it comes to medication adherence as well as anxiety or depression.

Patient reported outcome measures (PROMs) are not commonly explored in African research in general and previous, research on HRQoL in similar settings, has mainly focused on patients with human immunodeficiency virus (HIV) infection [55]. We therefore hope that our findings will add to the knowledge on HRQoL among a less researched patient group.

## Supplementary Information

Below is the link to the electronic supplementary material.

Supplementary file1 (JPEG 252 KB)

Supplementary file2 (DOCX 14 KB)

Supplementary file3 (DOCX 22 KB)

Supplementary file4 (DOCX 19 KB)

## Data Availability

Data is available upon reasonable request.
