# Supplementary figures and images for: Health-related quality of life among patients with rheumatoid arthritis in Zanzibar: a prospective cohort study
Source: Qual Life Res. 2025 May 7;34(7):2123–35. doi: 10.1007/s11136-025-03974-3 (PMC12182508; doi:10.1007/s11136-025-03974-3)

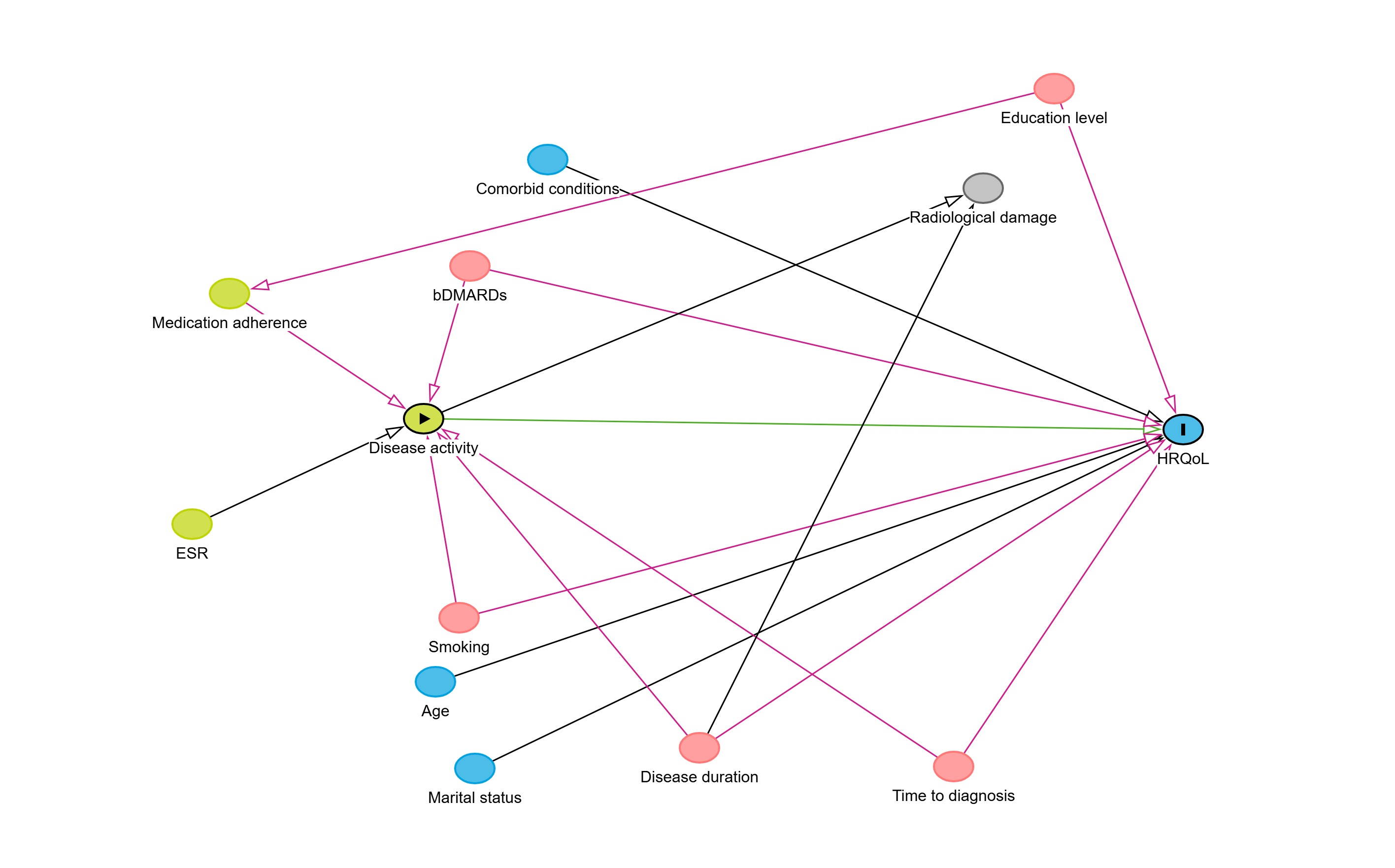

Supplement: Supplementary file 1 — Supplementary file1 (JPEG 252 KB) [file 11136_2025_3974_MOESM1_ESM.jpeg]
