# Supplementary material for: Health-related quality of life among patients with rheumatoid arthritis in Zanzibar: a prospective cohort study
Source: Qual Life Res. 2025 May 7;34(7):2123–35. doi: 10.1007/s11136-025-03974-3 (PMC12182508; doi:10.1007/s11136-025-03974-3)
Supplement: Supplementary file 2 — Supplementary file2 (DOCX 14 KB) [file 11136_2025_3974_MOESM2_ESM.docx]

Supplement 2

|  | mo | sc | ua | pd | ad |
| --- | --- | --- | --- | --- | --- |
| mo | 1.0000 |  |  |  |  |
| sc | 0.5331 | 1.0000 |  |  |  |
| ua | 0.6547 | 0.7281 | 1.0000 |  |  |
| pd | 0.4769 | 0.5306 | 0.5759 | 1.0000 |  |
| ad | 0.3831 | 0.3774 | 0.3082 | 0.3121 | 1.0000 |
