# Supplementary material for: Health-related quality of life among patients with rheumatoid arthritis in Zanzibar: a prospective cohort study
Source: Qual Life Res. 2025 May 7;34(7):2123–35. doi: 10.1007/s11136-025-03974-3 (PMC12182508; doi:10.1007/s11136-025-03974-3)
Supplement: Supplementary file 3 — Supplementary file3 (DOCX 22 KB) [file 11136_2025_3974_MOESM3_ESM.docx]

**Supplementary 2: Regression analysis of factors associated with utility scores in patients with rheumatoid arthritis**

| **Variable** | **Coefficient** | **Std. err.** | **t** | **95% CI** | ***p-*value** |
| --- | --- | --- | --- | --- | --- |
| Age | 0.010 | 0.007 | 1.35 | -0.005; 0.024 | 0.182 |
| **Sex** |  |  |  |  |  |
| Female | -1.047 | 0.589 | -1.78 | -2.225; 0.131 | 0.080 |
| **Marital status** |  |  |  |  |  |
| Married | -0.349 | 0.324 | -1.07 | -0.997; 0.300 | 0.287 |
| Divorced | -0.100 | 0.348 | -0.29 | -0.795; 0.594 | 0.774 |
| Widowed | -0.356 | 0.418 | -0.85 | -1.191; 0.479 | 0.398 |
| **Education level** |  |  |  |  |  |
| Secondary | 0.217 | 0.183 | 1.19 | -0.148; 0.583 | 0.239 |
| Tertiary | -0.055 | 0.258 | -0.21 | -0.572; 0.461 | 0.831 |
| No formal education | 0.347 | 0.258 | 1.35 | -0.168; 0.863 | 0.183 |
| **Household expenses (USD)** | -0.004 | 0.091 | -0.05 | -0.185; 0.177 | 0.962 |
| **Smoking history** |  |  |  |  |  |
| History of smoking | -0.120 | 0.555 | -0.22 | -1.228; 0.989 | 0.830 |
| **Disease duration** |  |  |  |  |  |
| ≥1 year | 0.350 | 0.249 | 1.41 | -0.147; 0.848 | 0.164 |
| **Time to diagnosis** |  |  |  |  |  |
| ≥1 year | -0.458 | 0.180 | -2.54 | -0.819; -0.098 | 0.013 |
| **Medication** |  |  |  |  |  |
| bDMARDs | -1.266 | 0.488 | -2.59 | -2.242; -0.29 | 0.012 |
| **Comorbid** |  |  |  |  |  |
| Single | 0.301 | 0.157 | 1.91 | -0.014; 0.615 | 0.061 |
| More than one | 0.135 | 0.202 | 0.67 | -0.269; 0.538 | 0.507 |
| **Radiological damage** |  |  |  |  |  |
| Yes | 0.022 | 0.156 | 0.14 | -0.290; 0.334 | 0.888 |
| **ESR** | 0.002 | 0.003 | 0.62 | -0.004; 0.007 | 0.538 |
| **CDAI** |  |  |  |  |  |
| Low | -0.215 | 0.173 | -1.24 | -0.560; 0.131 | 0.219 |
| Moderate | -0.709 | 0.191 | -3.72 | -1.090; -0.328 | 0.000 |
| High | -0.750 | 0.237 | -3.16 | -1.224; -0.276 | 0.002 |
