# Supplementary material for: Health-related quality of life among patients with rheumatoid arthritis in Zanzibar: a prospective cohort study
Source: Qual Life Res. 2025 May 7;34(7):2123–35. doi: 10.1007/s11136-025-03974-3 (PMC12182508; doi:10.1007/s11136-025-03974-3)
Supplement: Supplementary file 4 — Supplementary file4 (DOCX 19 KB) [file 11136_2025_3974_MOESM4_ESM.docx]

**Supplementary 3: Regression analysis of factors associated with visual analog scale scores in patients with rheumatoid arthritis**

| **Variable** | **Coefficient** | **Std. err** | **t** | **95% CI** | ***p-*value** |
| --- | --- | --- | --- | --- | --- |
| **Age** | 0.018 | 0.010 | 1.80 | -0.002 ; 0.039 | 0.077 |
| **Sex** |  |  |  |  |  |
| Female | -0.547 | 0.846 | -0.65 | -2.238 ; 1.144 | 0.521 |
| **Marital status** |  |  |  |  |  |
| Married | -0.212 | 0.466 | -0.46 | -1.143 ; 0.719 | 0.650 |
| Divorced | -0.364 | 0.499 | -0.73 | -1.361 ; 0.634 | 0.469 |
| Widowed | -0.269 | 0.600 | -0.45 | -1.468 ; 0.930 | 0.655 |
| **Education level** |  |  |  |  |  |
| Secondary | 0.143 | 0.261 | 0.55 | -0.379 ; 0.665 | 0.587 |
| Tertiary | -0.527 | 0.368 | -1.43 | -1.263 ; 0.209 | 0.157 |
| No formal education | 0.569 | 0.369 | 1.54 | -0.169 ; 1.308 | 0.128 |
| **Household expenses (USD)** | -0.394 | 0.120 | -3.29 | -0.634 ; -0.154 | 0.002 |
| **Smoking history** |  |  |  |  |  |
| History of smoking | -0.228 | 0.797 | -0.29 | -1.820 ; 1.364 | 0.776 |
| **Disease duration** |  |  |  |  |  |
| Above one year | -0.240 | 0.358 | -0.67 | -0.955 ; 0.474 | 0.504 |
| **Time to diagnosis** |  |  |  |  |  |
| 1 year & above | -0.532 | 0.251 | -2.12 | -1.033 ; -0.03 | 0.038 |
| **Year1medscoded** |  |  |  |  |  |
| bDMARDs | 0.731 | 0.699 | 1.05 | -0.665 ; 2.127 | 0.300 |
| **Comorbid** |  |  |  |  |  |
| Single | -0.030 | 0.224 | -0.13 | -0.479 ; 0.418 | 0.893 |
| More than one | -0.438 | 0.290 | -1.51 | -1.017 ; 0.141 | 0.136 |
| **Radiological damage** |  |  |  |  |  |
| Yes | -0.326 | 0.222 | -1.47 | -0.769 ; 0.117 | 0.147 |
| **ESR** | 0.002 | 0.004 | 0.43 | -0.006 ; 0.009 | 0.670 |
| **CDAI** |  |  |  |  |  |
| Low | -0.832 | 0.248 | -3.35 | -1.327 ; -0.336 | 0.001 |
| Moderate | -1.583 | 0.273 | -5.79 | -2.129 ; -1.037 | 0.000 |
| High | -1.686 | 0.341 | -4.95 | -2.367 ; -1.005 | 0.000 |
